# WHO target product profiles for TB preventive treatment

**DOI:** 10.5588/ijtld.21.0667

**Published:** 2022-04-01

**Authors:** S. Den Boon, C. Lienhardt, M. Zignol, K. Schwartzman, N. Arinaminpathy, J. R. Campbell, P. Nahid, M. Penazzato, D. Menzies, J. F. Vesga, O. Oxlade, G. Churchyard, C. S. Merle, T. Kasaeva, D. Falzon

**Affiliations:** 1 Global Tuberculosis Programme, World Health Organization (WHO), Geneva, Switzerland; 2 Unité Mixte Internationale TransVIHMI, Unité mixte internationale 233, Institut de recherche pour le développement, Unité 1175, Université de Montpellier, Institut de Recherche pour le Développement (INSERM), Montpellier, France; 3 Epidemiology and Population Health, Department of Infectious Disease Epidemiology, London School of Hygiene & Tropical Medicine, London, UK; 4 McGill International Tuberculosis Centre, McGill University, Montréal, QC, Canada; 5 MRC Centre for Global Infectious Disease Analysis; 6 Department of Infectious Disease Epidemiology, Faculty of Medicine, Imperial College London, London, UK; 7 Center for Tuberculosis, University of California, San Francisco, CA, USA; 8 Department of Global HIV, Hepatitis and Sexually Transmitted Infections Programmes, WHO, Geneva, Switzerland; 9 The Aurum Institute, Johannesburg, South Africa; 10 School of Public Health, University of Witwatersrand, Johannesburg, South Africa; 11 Special Programme for Research and Training in Tropical Diseases (TDR), Geneva, Switzerland

**Keywords:** tuberculosis, latent TB infection, research, prevention, drug treatment

## Abstract

**Background:**

The WHO has developed target product profiles (TPPs) describing the most appropriate qualities for future TPT regimens to assist developers in aligning the characteristics of new treatments with programmatic requirements.

**Methods:**

A technical consultation group was convened by the WHO to determine regimen attributes with greatest potential impact for patients (i.e., improved risk/benefit profile) and populations (i.e., reduction in transmission and TB prevalence). The group categorised regimen attributes as ‘priority’ or ‘desirable’; and defined for each attribute the minimum requirements and optimal targets.

**Results:**

Nine priority attributes were defined, including efficacy, treatment duration, safety, drug–drug interactions, barrier to emergence of drug resistance, target population, formulation, dosage, frequency and route of administration, stability and shelf life. Regimens meeting optimal targets were characterised, for example, as having superior efficacy, treatment duration of ≤2 weeks, and improved tolerability and safety profile compared with current regimens. The four desirable attributes included regimen cost, safety in special populations, treatment adherence and need for drug susceptibility testing in the index patient.

**Discussion:**

It may be difficult for a single regimen to satisfy all characteristics so regimen developers may have to consider trade-offs. Additional operational aspects may be relevant to the feasibility and public health impact of new TPT regimens.

## Abstract (fr)

**Contexte:**

L’OMS a développé des profils de produits (TPP) décrivant les caractéristiques les plus appropriées de futurs schémas de traitement preventif contre la TB (TPT), afin d’aider les fabricants à aligner les caractéristiques des nouveaux traitements sur les besoins programmatiques.

**Méthodes:**

Un groupe de consultation technique a été réuni par l’OMS afin de déterminer les caractéristiques des schémas dont l’impact pour les patients serait maximal (c.-à-d., amelioration du rapport bénéfices/risques), ainsi que pour les populations (c.-à-d., réduction de la transmission et de la prévalence de la TB). Le groupe a classé les caractéristiques des schémas en tant que « prioritaires » ou « souhaitables » et a défini pour chaque caractéristique les exigences minimales requises et les résultats optimaux.

**Résultats:**

Neuf caractéristiques prioritaires ont été définies, dont efficacité, durée du traitement, innocuité, interactions médicamenteuses, obstacle à l’émergence de résistances aux médicaments, population cible, préparation pharmaceutique, posologie, fréquence et voie d’administration, stabilité et durée de conservation. Les schémas satisfaisant les résultats optimaux ont par exemple été définis comme ayant une efficacité supérieure, une durée de traitement ≤2 semaines et un meilleur profil de tolérance et d’innocuité que les schémas actuels. Les quatre caractéristiques souhaitables étaient : coût du schéma, innocuité chez certaines populations spécifiques, observance thérapeutique et besoin de test de sensibilité aux antituberculeux chez les patients index.

**Conclusion:**

Un seul et unique schéma peut avoir du mal à satisfaire toutes les caractéristiques. Les fabricants pourraient donc être amenés à faire des compromis. D’autres facteurs opérationnels pourraient être pertinents quant à la faisabilité et à l’impact des nouveaux schémas de TPT sur la santé publique.

TB is a major yet preventable global health problem, with an estimated 9.9 million new cases worldwide in 2020 that resulted in more than 1.3 million deaths.^1^ A quarter of the global population is estimated to be infected with *Mycobacterium tuberculosis*.^2,3^ All people who are infected but do not have the disease have no signs or symptoms and are not contagious, although they are at risk of progression from infection to disease – at which time they can contribute to disease transmission. On average, 5–10% of people who are infected will develop disease over the course of their lives, with the highest risk in the first year after infection.^4,5^ People living with HIV are also at high risk of developing TB disease after infection. People at higher risk of developing TB disease include household contacts of infectious TB patients (particularly children <5 years of age), and people living with HIV (PLHIV), as well as people with the following conditions: those on anti-TNF treatment, those receiving dialysis, or preparing for an organ or haematological transplant, patients with silicosis. These also include health workers, the incarcerated, immigrants from high TB burden countries, homeless people and people who use drugs. All of these are eligible for systematic testing for TB infection and TB preventive treatment (TPT).^6^ The WHO End TB Strategy calls for 90% TPT coverage among PLHIV and close contacts of infectious TB patients by 2035.^7^ In September 2018, the United Nations High-Level Meeting on TB further emphasised the importance of strengthening implementation of TPT, with the goal of 30 million people receiving TPT by 2022.^8^

TPT has been available for more than 60 years. However, despite strong scientific evidence of its effectiveness, its uptake and scale-up in many high-burden countries have been slow for several reasons: long duration and potential toxicity of treatment (warranting targeted use to ensure a more favourable risk-benefit ratio), insufficient clinician conviction of the benefits of TPT, difficulties with regimen adherence and fear of emergence of drug resistance, and operational aspects such as product stability and cost.^9–11^ In addition, tests of TB infection are currently not able to predict risk of progression from infection to disease and this prevents targeted TPT beyond the high-risk groups mentioned above.^12^ The development and evaluation of novel and shorter regimens with reduced toxicity and improved toler-ability profile compared to current treatments is essential to ensure wider-scale implementation.^6, 13–15^

In this context, the WHO decided to develop target product profiles (TPP) to identify product attributes to be considered when investigating and developing novel TPT regimens and to ensure alignment with characteristics matching national programme requirements.^16^ It should be noted that target products are either an individual medicine or a combination of medicines. The target audience for this TPP includes innovating pharmaceutical companies, academia, research institutions, product development partnerships, non-governmental and civil society organisations and donors.

## Methods

Building on a process initiated by the WHO Task Force for new TB drugs and regimens that developed target regimen profiles for TB treatment,^17^ we established a technical consultation group with a wide range of experts including scientists, clinicians, TB programme managers, public health specialists, donors and civil society representatives (see Acknowledgements for the full list of experts). All experts made a declaration on potential interests and these were reviewed and managed by the WHO; a statement on Conflict of Interest is available in the WHO TPP document.^16^ The group consisted of 44 individuals who met for one day in Montreal, QC, Canada in September 2019 to develop the scope or “use case scenarios” under which an optimal TPT regimen would operate (Table 1).

We subsequently performed a scoping review, providing an overview of the different TPT treatments available or under development and prepared an initial working draft of product attributes and potential targets.^18^ This draft was then refined through several rounds of virtual discussion and consensus seeking with the expert group. In parallel, two complementary studies were conducted to inform the discussion: 1) a modelling study to identify regimen attributes that maximised the impact on population health in different epidemiological settings, and 2) a cost-effectiveness analysis that estimated health system costs associated with the different scenarios considered in the modelling study (these modelling studies will be published separately). Following the preparation of the draft document, it was made available for public comment for a month on the WHO website, specifically inviting relevant stakeholders and interested parties to provide feedback.

The main findings from the discussions and additional studies were summarised, with a set of regimen attributes representing the most important performance and operational characteristics for TPT. Consensus was obtained on the selection of attributes that were considered essential for public health impact; these were designated as “priority” attributes, while others deemed less essential were termed “desirable”. While priority attributes constitute grounds for “go/no-go” decision, desirable attributes could be considered for trade-offs. For example, if a new regimen were to be well-tolerated or have strong efficacy, a trade-off could be justified in a desirable area such as the number of drugs in the regimen.

For each attribute, we additionally defined minimum and optimal requirements through a consensus-seeking process: 1) “minimum requirements” represent target characteristics of a candidate regimen that are the same or better than the current standard-of-care and are considered an acceptable minimum for global health impact. They provide the basis for critical decisions needed throughout the development and assessment process. 2) “Optimal requirements” specify the performance and use characteristics of an "ideal" product, needed to broaden and accelerate the global health impact. It is expected that the resulting future TPT regimens will have at least all the minimum requirements of the priority attributes, and as many of the desirable attributes as possible.

## Results

Thirteen attributes were identified and listed in Table 2 (priority attributes) and Table 3 (desirable attributes). For each attribute, annotations are provided to clarify the rationale for selecting the minimum and optimal targets.

### Priority attributes

#### Indication

A new TPT should be efficacious against all *M. tuberculosis* strains harboured by an individual and be effective regardless of the resistance profile of the strain. However, given that most infected people harbour bacilli that are susceptible to rifampicin (RIF), the “minimum” target should be a TPT regimen effective against RIF-susceptible TB for contacts of infectious TB patients and PLHIV, who are priority groups for TB prevention.^6^ Under the “optimal” scenario, the TPT regimens should treat TB infection in *all* individuals, regardless of the resistance profile of the *M. tuberculosis* strains they host (i.e., contacts of patients with both drug-susceptible and drug-resistant TB).

#### Efficacy

In clinical trials, the efficacy of treatment for TB infection is usually defined as the relative reduction in incidence of TB disease after treatment completion (usually 2–5 years) compared to untreated persons. The expected duration of protection, however, depends on many factors, including patient characteristics (i.e., age, sex and host immune response capacity) and local epidemiology and setting (i.e., high or low TB transmission areas, with varying contributions of reinfection vs. reactivation).^19^ Under the “minimum” target, a regimen should have non-inferior efficacy to the current standard-of-care (e.g., 6H or 3HP, i.e., 6 months of isoniazid [INH] or 3 months of INH and rifapentine [RPT]) while an “optimal” regimen has superior efficacy.

#### Duration of treatment

Meta-analyses have shown that shorter regimens are generally associated with higher treatment completion rates in addition to improved adherence.^19^ Long-acting, extended-release, oral or injectable formulations to be administered intramuscularly or subcutaneously could minimise erratic adherence and treatment interruptions.^20^ A new regimen for TPT should have a treatment duration of <3 months under the “minimum” scenario, and a duration of ≤2 weeks under the “optimal” regimen.

#### Safety and tolerability

As TB infection itself is not associated with health effects or symptoms, safety and tolerability of regimens are tightly linked to the likelihood of treatment adherence. The most important risk associated with current TPT regimens is hepatotoxicity, but other adverse events should be considered, including cutaneous reactions, hypersensitivity reactions, gastrointestinal intolerance, neurotoxicity and cardiotoxicity, as these can lead to temporary or permanent cessation of treatment and could threaten health if severe. The toxicity risk of current TPT is the main reason why it is only indicated in people with a higher risk of progression than the general population. Conversely, a safer regimen could be given to people with lower progression risk. This would make TPT easier to implement, as it would simplify assessment of eligibility (e.g., not requiring testing for TB infection) and toxicity monitoring while on treatment. Furthermore, more front-line providers will be able to initiate and monitor its use. Therefore, “minimum” target indicates the need for a better safety profile compared to the current standard-of-care, while the “optimal” target requires a better safety profile than the current safest preventive treatment (i.e., 3 months weekly RPT + INH, 4 months daily RIF monotherapy or 1 month daily RPT + INH), with no adverse events leading to treatment cessation. In addition, there should be no requirement for active clinical or laboratory monitoring for drug toxicity.

#### Drug-drug interactions and metabolism

Targets are provided for the most common and important drug–drug interactions observed to date, such as with antiretrovirals when administered to PLHIV. Other concurrent conditions should be considered as well, especially those requiring long-term treatment (diabetes, hypertension, solid organ transplant, rheumatological diseases), as well as parasitic diseases (e.g., malaria). Drug–drug interactions are one of the barriers to the use of rifamycins. It might not be possible to avoid all drug–drug interactions, and the “minimum” target therefore specifies that they should be manageable with simple clinical algorithms. The “optimal” target specifies that there should be no need for dose adjustment if TPT is given with any other medication and that TPT can be used safely with any other drug.

#### Barrier to emergence of drug resistance

A low barrier to the emergence of drug resistance means that relatively few mutations may lead to drug resistance to the medicines used. The potential emergence of INH resistance has long been an argument against provision of TPT in programmes, although there is strong evidence to suggest that there is no significant risk when TB disease is appropriately excluded.^21,22^ Novel TPT regimens may permit a rate of in-vitro resistance conferring mutations that is lower than with RIF, so that there is a high barrier to emergence of drug resistance, and this barrier to resistance should also apply if repeated TPT courses may be necessary, e.g., in PLHIV or in high-burden settings.

#### Target population

The “minimum” targets are priority populations, including PLHIV, household and other close recent contacts of persons with infectious TB (including neonates and young children), as well as other at-risk populations, such as patients who are initiating anti-TNF treatment, have chronic renal failure, are preparing for organ or haematological transplant or who have silicosis.^6^ However, availability of safe regimens would allow broadening the target population to include people at much lower risk of TB disease, such as general populations in moderate-or high-incidence settings; therefore, this is considered the “optimal” target.

#### Formulation, dosage, frequency and route of administration

In case of oral regimens, there should be a maximum intake of one daily dose, and in the “optimal” target regimens, weight adjustment should not be required. Paediatric forms (dispersible, scored tablets, with good palatability) need to be available. If the regimen consists of two or more oral drugs, fixed-dose combinations are optimal to facilitate use by all targeted recipients of TPT. For PLHIVs, fixed-dose combinations could combine TPT medicines with antiretrovirals or cotri-moxazole. With regard to long-acting formulations, the “minimum” target specifies injections with or without oral lead-in no more than once monthly, or a single dissolvable implant for the complete course of therapy, while the “optimum” target specifies a single injection of long-acting formulation without oral lead-in, to be given once or twice; or alternatively a single dissolvable implant with long-lasting protection.^20^

#### Stability and shelf-life

Currently available treatments are stable for at least 24 months in standard conditions. At least similar shelf life would be expected for the next generation of TPT under the “minimum” scenario, and a shelf-life of ≥5 years in the “optimum” scenario. As TPT would have to be provided in high TB burden countries that are likely to be hot and humid, the medicines must be stable in all climate zones and, preferably, not require a cold chain.

### Desirable attributes

#### Cost of regimen

TPT costs should be compatible with wide access and scale-up. Costs for the programmatic management of TPT include the cost of the medication itself, but also health system and patient costs associated with administration and monitoring during treatment.

An improved regimen may decrease the costs to programmes and patients if it is shorter, safer, better tolerated, requires minimal to no monitoring or testing for TB infection to balance risk and benefits, and reduces the need for management of adverse events or toxicity. The medication price and the associated resources required to provide the regimen should be evaluated while carefully considering principals of equity, non-discrimination and transparency, with the goal of affordable access for all, ensuring that vulnerable and marginalised individuals do not bear disproportionate costs.

#### Special populations

Novel TPT regimens should be safe for women of reproductive age, and pregnant and lactating women.^14,23^ There must be no increased risk of major birth defects based on pre-clinical studies and pharmacokinetics and safety should be demonstrated. Antenatal and postnatal care and care during delivery is necessary to reduce the risk of adverse maternal and pregnancy outcomes, with minimal risk for negative health outcomes in exposed infants. In children, pharmacokinetics and safety studies will be required in both the minimum and optimum scenarios, but efficacy trials in this population are not necessarily required. TB regimen developers should consider initiating pharmacokinetics and safety studies in all paediatric age groups for drugs that show promising efficacy and safety in Phase 2A trials in adults, as this information would be useful to inform decision-making for TPT.^13^

#### Treatment adherence and completion

Adherence to treatment is a complex behaviour that is influenced by many factors such as personal motivation, beliefs about health, risks and benefits from treatment, and comorbidities. Interventions to support adherence that are used for TB treatment could be applied to TPT, including digital technologies.^24^ For the minimum target, most patients on a new regimen should be able to complete self-administered therapy. For the optimal target, self-administered therapy should be the standard in all populations.

#### Drug susceptibility testing in the index TB patient

In an optimal scenario, the new treatment regimen should be usable in all epidemiological conditions and settings, regardless of drug resistance burden, without any need for drug susceptibility testing (DST) of the index patient. In the minimum scenario, a single, rapid, molecular test should be available to assess the DST profile of the bacilli population in an index patient and confirm the suitability of the TPT regimen. For currently available regimens, these molecular tests should be able to test for RIF susceptibility, but for future new regimens this may also require other DST.

## Discussion

The TPPs presented here include all attributes that are considered essential for novel TPT. However, it might be difficult to satisfy all attributes in a single regimen. Regimen developers might thus have to consider trade-offs, such as increased efficacy and safety at the expense of treatment duration, or increasing dosing frequency to reduce significantly the duration of treatment. For an infectious disease such as TB, with a large global burden and continuing person-to-person transmission, the efficacy of new TPT regimen(s) will depend heavily on operational factors that also affect a regimen's ability to fulfil its role and the scale at which it can be implemented (e.g., access to diagnostics, capacity to rule out TB disease, access to new treatments, education and commitment of health care workers, patients and contacts). While the TPPs indicate the attributes to be considered at the developmental and investigational stage, these should not be disconnected from the factors that heavily influence implementation in the larger context of TB programme activities, as these would contribute to the overall success of any new regimens.

Indeed, a number of operational factors need to be considered, such as the diagnosis of TB infection, DST of index or source patients, acceptability of and adherence to treatment, as well as the need for monitoring during treatment. Current WHO guidelines stipulate that “testing for TB infection by tuberculin skin test or interferon-gamma release assay is not a requirement for initiating preventive treatment in PLHIV or child household contacts aged <5 years,” because it would otherwise pose a barrier to starting these high-risk groups on TPT.^6^ However, for other target groups, e.g., adults, adolescents and children aged ≥5 years who are household contacts of bacteriologically confirmed pulmonary TB patients, or migrants arriving from endemic areas, testing for TB infection is desirable before starting TPT. The strategy therefore differs according to the population groups to be considered, and various use case scenarios may be developed for global use and scale-up of treatments. It is hoped that cheap, rapid, accurate, point-of-care diagnostic tests for TB infection will become available for further refinement of the TB prevention strategy. An efficacious TPT, if coupled with a test to identify those at highest risk for disease progression, would be an important step to accelerate progress towards TB elimination.^24^ Development of new TPT should thus include consideration of diagnostic technologies for identifying those at greatest risk of progression to TB disease. In the absence of these, and in every situation in which TPT is indicated and considered, it is imperative that TB disease be formally ruled out, as inadvertent use of TPT in people with TB disease may generate drug resistance. Furthermore, close monitoring is needed to ensure early identification of people who may develop TB disease despite receiving TB preventive treatment.^6^ However, the need to test individuals before initiating TPT will conceivably diminish as less toxic and more convenient regimens become available, making them safe to administer to larger segments of the population at lower risk of progression than conventional target populations. Such an approach of mass drug administration has been successfully implemented for selected neglected tropical diseases.^25^ This is an important consideration in settings aiming to fully eliminate TB or where TB incidence remains high despite mitigation efforts.

As for treatment acceptability, it is essential to keep in mind that TPT is offered to individuals who are otherwise healthy and do not necessarily consider themselves affected by any specific illness. Therefore, preventive treatment should be extremely safe and should not impose additional health concerns or otherwise disrupt the activities of individuals who agree to take TPT. As shorter regimens are associated with higher treatment completion rates, any potential gain in completion from a shorter duration must not be offset by poorer safety or tolerability.^26^ Furthermore, any regimen that appears to be promising as a future TPT should be evaluated carefully for safety and tolerability in the same populations as those likely to receive it. Therefore, as part of clinical development, trials should be performed to define the benefits and harms of TPT in groups at risk, including PLHIV, children, pregnant and lactating women, recent contacts of infectious TB patients, and people who are immune-suppressed.^14^

Finally, consideration should be given to the larger health context in which TPT would be used. TPT is a key intervention to reduce TB incidence but should be used in association with efficient measures to reduce TB infection and disease transmission, including early diagnosis and treatment of TB disease, reduction of comorbidities (such as diabetes), and health risks (such as smoking and alcohol use). Even with TPT regimens that are shorter, safer, more convenient and feasible, a multipronged approach is likely to remain necessary. In high TB incidence settings with continuous TB transmission and concurrent HIV epidemics, the protective effect of TPT may be transient, given the potential for re-infection; treatment would nonetheless be particularly beneficial in vulnerable groups, such as young household contacts of TB patients or PLHIV. The impact of TPT will depend largely on the capacity to initiate and sustain targeted campaigns for the prevention of TB in these high-risk groups. This should complement sustained provision of ART, which is a highly effective measure against TB and other opportunistic infections in PLHIV.^27^ Conversely, in low TB incidence settings with minimal TB transmission, the protection provided by TPT is more durable, given the limited risk of reinfection. In deciding whether to recommend TPT, clinicians should weigh the probable benefits and harms of treatment (once TB disease has been ruled out), considering the risk of subsequent progression to disease and the potential toxicity of therapy, which requires consideration of the patient's age and comorbid conditions. Should an incidence-reducing vaccine against TB become licensed and widely available within the timeline of the End TB Strategy, synergies between vaccine and enhanced TPT options may be envisaged.^28^

In conclusion, the new WHO TPPs for TPT represent an important statement by global stake-holders about the type of regimens that should be harnessed, to deliver upon global targets. The use of this TPP for TPT is critical to ensure targeted research and development efforts, and allocation of resources to innovation that is most likely to generate health impact. Developing safer, shorter, better and highly accessible regimens for TPT will be critical to achieving a world free of TB.

## Figures and Tables

**Table 1:** Use case scenario

To shape the overall use of TPT, an essential use case scenario was developed, under which an optimal TPT regimen should: Be indicated for treatment of all individuals and age groups with TB infection at risk of developing TB disease, irrespective of HIV status; Be safe, tolerable and efficacious in individuals of all ages and subpopulations (including neonates, infants and young children, women of reproductive age, pregnant and lactating women) and for patients with a wide range of comorbidities, including HIV infection, other infectious or tropical diseases and chronic diseases; Be free of drug-drug interactions, specifically with antiretrovirals, drugs metabolised by P450 liver enzymes, pro-arrhythmic QT-prolonging drugs, contraceptive medicines; Be delivered exclusively orally with a simple dosing schedule (preferably once daily, with no food restriction, no requirement for weight adjustment and in a fixed-dose combination) and be child-friendly (e.g., dispersible, scored and palatable formulations with few pills); injectable formulations may be considered for long-acting formulations with significant benefit in extending protection and reducing frequency of administration; Be affordable and accessible, particularly in low- and middle-income countries; Contain medicines that may be prescribed in decentralised settings; Be simple to use and easy to monitor through simple checks; Be usable in all conditions and settings, particularly where there is a moderate-to-high likelihood of rifampicin-resistant TB transmission and delivered without requiring testing of susceptibility profile for the source case

TPT = TB preventive treatment.

**Table 2:** Priority attributes for TB preventive treatment

Attribute	Minimum*	Optimal†
Indication	The regimen is indicated for the treatment of TB infection to prevent development of TB disease in at-risk individuals as defined in current WHO guidelines	The regimen is indicated for the treatment of TB infection to prevent development of TB disease in all individuals recognised as being at risk of TB disease, regardless of the drug susceptibility profile of the harboured bacillary population
Efficacy	A regimen with efficacy not inferior to the current standard of care for treatment of TB infection (e.g., 6H or 3HP)‡	A regimen with efficacy superior to the current standard of care regimen for treatment of TB infection, leading to lifetime protection in areas with low risk of re-infection
Duration of treatment administration	<3 months	≤2 weeks
Safety and tolerability	Safety: Incidence and severity of adverse events better than the current standard of care treatment. Requirement of no more than monthly clinical monitoring and no laboratory monitoring for drug toxicity necessary, except for special populations (e.g., those with pre-existing kidney or liver disease, diabetes). The target product should not require any additional medication to allay toxicity (e.g., pyridoxine in IPT) Tolerability: The frequency of adverse events leading to treatment cessation should be no worse than with current paired isoniazid and rifamycin regimens (e.g., 3HP, 3HR, 1HP‡)	Safety: Incidence and severity of adverse events better than the current safest treatment (i.e., 4R and 3HP regimens‡). No requirement for active clinical monitoring or for laboratory monitoring for drug toxicity and preferably no requirement for additional monitoring or encounters for special populations (e.g., those with pre-existing liver disease, diabetes). The target product should not require any additional medication to allay toxicity (e.g., pyridoxine in IPT) Tolerability: No adverse events leading to treatment cessation
Drug−drug interaction and metabolism	Can be used safely with any other medication with minimal dose adjustment, particularly with current first- and second-line ART, opioid substitution therapies, hormone-based contraceptives and directly acting antivirals to treat viral hepatitis	No dose adjustment when given with any other medication and can be used safely with any other drug
Barrier to emergence of drug resistance (propensity to develop resistance, generation of cross-resistance)	Potential for acquisition of resistance is no worse than with current regimens and current methods of excluding TB disease	Rate of in-vitro mutations conferring resistance is lower than with rifampicin Fewer than two gene targets linked to development of resistance No cross-resistance with existing drugs
Target population	Populations with established high risk of progression to TB disease, e.g., HIV-infected adults, adolescents and children aged ≥12 months (with unknown or a positive tuberculin skin test) regardless of ART use; household contacts of people with bacteriologically confirmed pulmonary TB; patients with immunosuppressive conditions such as initiation of anti-TNF treatment, chronic renal failure treated with dialysis, preparation for organ or haematological transplant and silicosis In all these situations, TB disease must be formally ruled out	All individuals in all age groups at risk of TB disease, irrespective of HIV status, whether living in countries with high, medium or low TB incidence, regardless of the drug susceptibility profile of the harboured bacilli population, in whom TB disease has been formally ruled out
Formulation, dosage, frequency and route of administration	Formulation to be oral, with once daily intake as a maximum for all drugs in the regimen, including paediatric forms (dispersible, scored tablets, palatability) If long-acting formulation: injection with or without oral lead-in no more than once monthly Single dissolvable implant for complete course of therapy	Formulation to be oral, without a requirement for weight adjustment, including paediatric forms (dispersible, scored tablets). Ideally, a single pill per day or week for the duration of treatment (depending on daily or weekly formulation) No specific food requirements Single injection of long-acting formulation without oral lead-in to be given once or twice or a single dissolvable implant with long-lasting protection
Stability and shelf life	Oral regimen: Stable to heat, humidity and light, with a shelf life for all drugs ≥2 years. No cold chain required Injectable: stable in all climate zones. If cold chain required, to be compatible with current vaccine cold chain requirements (2–8°C)	Oral regimen: Stable to heat, humidity and light, with a shelf life for all drugs ≥5 years. No cold chain required Injectable: stable in all climate zones, and no cold chain required

* The minimal target should be considered a potential “go” or “no go” decision point for the given “priority attribute”.

† The optimal target should have a broader, deeper, quicker global health impact.

‡ 1HP=1 month daily rifapentine plus INH; 3HP=3 months weekly rifapentine plus INH; 3HR=3 months daily rifampicin plus INH; 4R=4 months daily rifampicin monotherapy; 6H = 6 months daily INH monotherapy; 9H = 9 months daily INH monotherapy. IPT = INH preventive therapy; ART =antiretroviral therapy.

**Table 3:** Desirable attributes for TB preventive treatment

Attribute	Minimum	Optimal
Cost of regimen	Projected cost of the drug(s) or treatment courses should be compatible with wide access	Projected cost of the drug(s) or treatment course should be compatible with wide access and scale-up
Special populations	For women of reproductive age, pharmacokinetics and safety studies support use of the regimen with minimal dose adjustment There must be no foetal risk profile in preclinical data The drugs can be taken safely during breastfeeding and are compatible with common forms of hormone-based birth control for women of reproductive age who wish not to become pregnant Safe and tolerable for patients with comorbid conditions	For women of reproductive age and pregnant women, pharmacokinetics and safety studies support use of the regimen without dose adjustment There must be no foetal risk profile in preclinical data The drugs can be taken safely during breastfeeding and are compatible with common forms of hormone-based birth control for women of reproductive age who wish not to become pregnant Safe and tolerable for patients with comorbid conditions
Treatment adherence and completion	At least as good adherence and likelihood of treatment completion as with existing recommended short-course regimens (4R, 3HP)* Suitable for self-administration in all populations (not for long-acting formulation)	Better adherence and likelihood of completion than with existing recommended short-course regimens Suitable for self-administration in all populations (not for long-acting formulation)
Need for drug susceptibility testing	Drug susceptibility test available for the index TB patient when required	No need to test index TB patient
